# Glycerophospholipids in ALS: insights into disease mechanisms and clinical implication

**DOI:** 10.1186/s13024-025-00876-3

**Published:** 2025-07-26

**Authors:** Thibaut Burg, Ludo Van Den Bosch

**Affiliations:** 1 Departement of Neuroscience and Leuven Brain Institute (LBI), KU Leuven - University of Leuven, Campus Gasthuisberg O&N5, Herestraat 49, BP 602, Leuven, 3000 Belgium; 2 Laboratory of Neurobiology, Center for Brain & Disease Research, VIB, Leuven, 3000 Belgium

**Keywords:** Amyotrophic lateral sclerosis, Neurodegeneration, Motor neuron, Lipid metabolism, Glycerophospholipid

## Abstract

Amyotrophic lateral sclerosis (ALS) is a devastating neurodegenerative disease affecting the adult motor system, with no effective treatments available. Despite extensive research efforts, the exact pathological cascade leading to progressive motor neuron degeneration remains elusive. Recent evidence highlights significant modifications in lipid metabolism during ALS progression, even before the onset of motor symptoms. Glycerophospholipids, the primary components of cellular membranes, are frequently altered in ALS patients and models. These lipids not only play a structural role in membranes, but also contribute to cellular metabolism, signaling pathways, and cell type-specific processes such as neuronal transmission and muscle contraction. In this review, we discuss glycerophospholipid physiological functions in the motor system and review recent studies demonstrating their alterations and the possible underlying pathological mechanisms in ALS. Furthermore, we discuss challenges emerging from studying lipid alterations in neurodegeneration and evaluate the therapeutic potential of glycerophospholipids.

## Background

Amyotrophic lateral sclerosis (ALS) is a neurodegenerative disease characterized by the degeneration of upper motor neurons (UMNs), located in the cerebral cortex, and lower motor neurons (LMNs), located in the brainstem and spinal cord [1]. ALS affects about 1–3 per 100,000 individuals annually, while the lifetime risk is 1/350 for men and 1/400 for women [2, 3]. Symptom onset mostly occurs during middle adulthood (50–65 years), and clinical manifestations include fasciculations, hyperreflexia, spasticity, muscle wasting, and progressive paralysis. In about 50% of ALS cases, mild to severe cognitive impairments are associated with the disease, highlighting the clinical overlap between ALS and frontotemporal dementia (FTD) [1]. Patients typically die within 2–5 years after the initial diagnosis mostly due to respiratory failure. Nonetheless, the type of symptoms, the age of onset, and the rate of disease progression can be highly variable [1].

The etiology is still not fully elucidated. About 10% of patients have a family history, while the remaining 90% of patients are considered as suffering from sporadic ALS as no known family members were diagnosed with the same disease. More than 30 genes have been linked to ALS, with the four most common genetic causes being mutations in *SOD1*, *TARDBP*, and *FUS* and hexanucleotide repeats in *C9ORF72* [4]. Similar to other neurodegenerative diseases, a multitude of pathogenic mechanisms have been suggested [5]. These comprise aberrant RNA metabolism, impaired protein homeostasis, axonal transport defects, excitotoxicity, neuroinflammation, mitochondrial impairments, oxidative stress, glial dysfunction, among several others [6]. All these pathological mechanisms ultimately culminate in a final common pathway contributing to the selective and progressive degeneration of motor neurons (MNs).

The diagnosis of ALS remains challenging and relies on an integrative approach with clinical examination, medical history, and exclusion of confounding diseases. As a consequence, 10–16 months are still necessary to establish a definite diagnosis. As a timely therapeutic intervention might be needed to have a clinical effect, an early diagnosis is crucial [7, 8]. Promising biomarkers derived from clinical, neurophysiological, neuroimaging, and genetic studies have emerged [9]. However, their sensitivity and specificity remain elusive. Neurofilaments are one of the rare biomarkers used in a clinical practice, with NF-L being the most robust one [10]. Despite intense research efforts and over two hundreds clinical trials, clinical translation has been poor [11]. Riluzole and Edaravone remain the only non-curative drugs available for ALS, with a limited benefit for the patient regarding an increase in quality of life and/or survival [12]. Although antisense oligonucleotides (ASOs) treatments are promising, especially Tofersen (FDA and EMA approved) and Jacifusen (Phase 3 clinical trial), they only concern a small proportion of familial ALS patients with mutations in specific genes [13]. As a consequence, the ALS community urgently needs new strategic approaches to discover innovative biomarkers for diagnosis and prognosis purposes and to identify reliable therapeutic targets.

Recent technological advances in mass spectrometry-based lipidomics have opened new avenues to study profound lipid metabolism alterations in patients suffering from neurodegenerative diseases such as Alzheimer’s disease, Parkinson’s disease, and peripheral neuropathies [14, 15]. Lipidomics generates powerful tools for understanding the variations of thousands of individual lipid species, allowing the identification of biomarkers and mapping of altered metabolic pathways [16]. Recent efforts have led to the development of the Neurolipid Atlas (https://neurolipidatlas.com/), a comprehensive open-access resource designed to collect and characterize lipidomic datasets across diverse brain regions, cell types, genetic mutations, neurodegenerative diseases, and model organisms. This initiative aims to advance our understanding of lipid metabolism alterations associated with neurodegeneration and to provide valuable insights into disease mechanisms [17].

Accumulating evidence suggests that lipid homeostasis is altered in ALS and plays an active role in disease onset and progression [18, 19]. However, additional studies are needed to understand the exact underlying mechanisms. In ALS patients, lipid-related biological parameters strongly predict progression and survival, while their dysregulation arises before motor symptoms onset [20–23]. Lipid defects have been observed in brain and spinal cord tissues, in skeletal muscles, in cerebrospinal fluid (CSF), and in blood samples. Several lipid classes are altered in ALS, including glycerophospholipids (GPLs), sphingolipids, cholesterol, and free fatty acids. Although GPLs account for more than 45% of the total dry weight of the brain [24] and as alterations are commonly observed in neurodegenerative diseases [18], GPLs received less attention compared to cholesterol or sphingolipids in ALS.

**Fig. 1:**
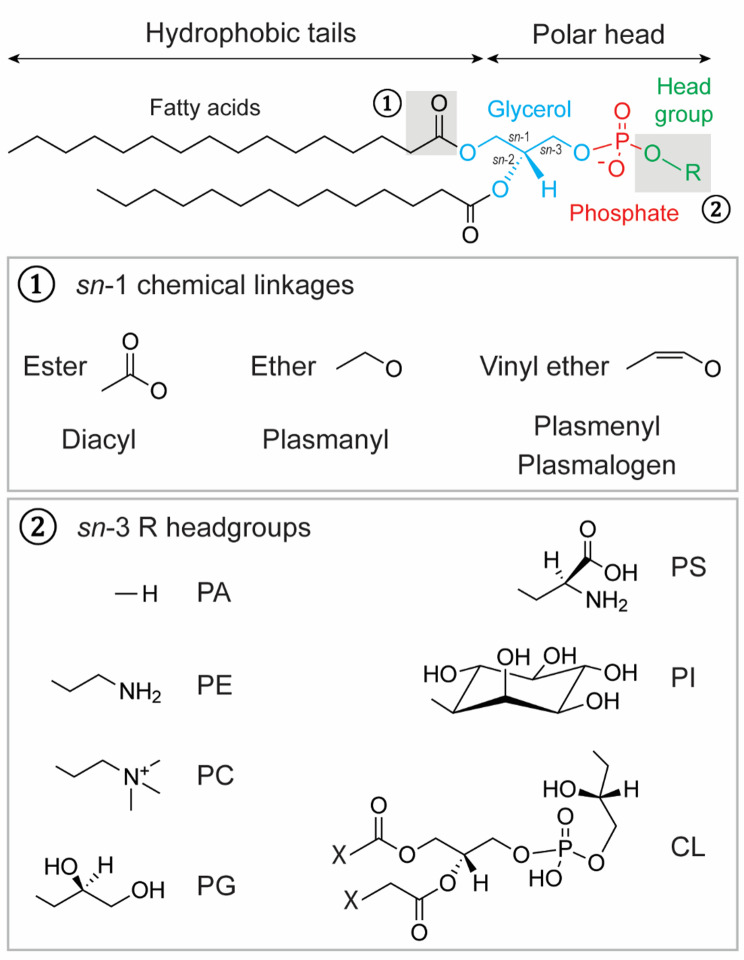
Structural and chemical diversity of GPLs. The glycerol backbone is esterified by fatty acids at the *sn*-1 and *sn*-2 positions and by phosphoric acid at the *sn*-3 position (**1**). The fatty acids can be replaced by fatty alcohols linked with an ether or vinyl ether bond. The different lengths and degrees of saturation of the fatty acids/alcohols further increase the diversity of GPLs (**2**). The phosphoryl group is linked to a polar head group with different chemical properties. CL: cardiolipin, PA: phosphatidic acid, PC: phosphatidylcholine, PE: phosphatidylethanolamine, PG: phosphatidylglycerol, PI: phosphatidylinositol, PS: phosphatidylserine

GPLs are structurally and biologically essential molecules, comprising 65–85% of total lipids (Table 1), that generate the complexity of membranes and participate in nearly all cellular processes [25]. Their numerous biological roles include the regulation of protein functions, vesicle trafficking, membrane fluidity, and signal transduction, among several others. As a consequence, GPLs should not be considered as passive structural building blocks, but rather as active biomolecules. GPLs comprise a glycerol backbone esterified at the *sn*-3 position with phosphoric acid and at the *sn*-1 and *sn*-2 positions with acyl chains (Fig. 1). This chemical structure confers amphipathic properties to GPLs. The hydrophilic charged head group forms the polar end, facing out towards the extracellular and intracellular sides of the membrane, while non-charged hydrophobic acyl chains form the non-polar end of GPLs. The wide range of GPL functions is due to the impressive diversity of their molecular species, created by the varying combinations of head groups and acyl chains (Fig. 1). According to the LIPID MAPS classification, GPLs are organized in 20 classes, grouping thousands of individual molecular species [26]. The relative abundance of lipid classes can considerably vary at the cellular and organellar level (Table 1). Hence, GPL concentrations are tightly regulated both spatially and temporally, maintaining homeostasis within narrow limits [27]. Aging and neurodegeneration can particularly affect this fragile equilibrium.

**Table 1:** Lipid composition of a representative mammalian cell and the major cell types of the central nervous system. Data are averaged from various references [17, 28, 197, 253]

% of total lipid		Representative cell	Neuron	Astrocyte	Microglia	Oligo- dendrocytes
Glycerophospholipids						
	PC	45–55	40	40	30	30
	PE	15–25	15	20	20	20
	PI	10–15	2	5	2	5
	PS	5–10	2	8	5	3
	PA	1–2	< 1	< 1	< 1	< 1
	PG	< 1	< 1	< 1	3	< 1
	CL	2–5	< 1	< 1	< 1	< 1
Sphingolipids		5–15	2	10	20	10
Sterol Lipids		10–20	40	25	25	35

This review summarizes the basics of GPL metabolism and its physiological roles, particularly in the motor system. Furthermore, we discuss GPL implications in ALS, particularly in the underlying pathological mechanisms, and will focus on the potential to develop new therapeutic strategies based on this knowledge.

### GPL metabolism in physiological condition

#### Biosynthetic pathways

**Fig. 2:**
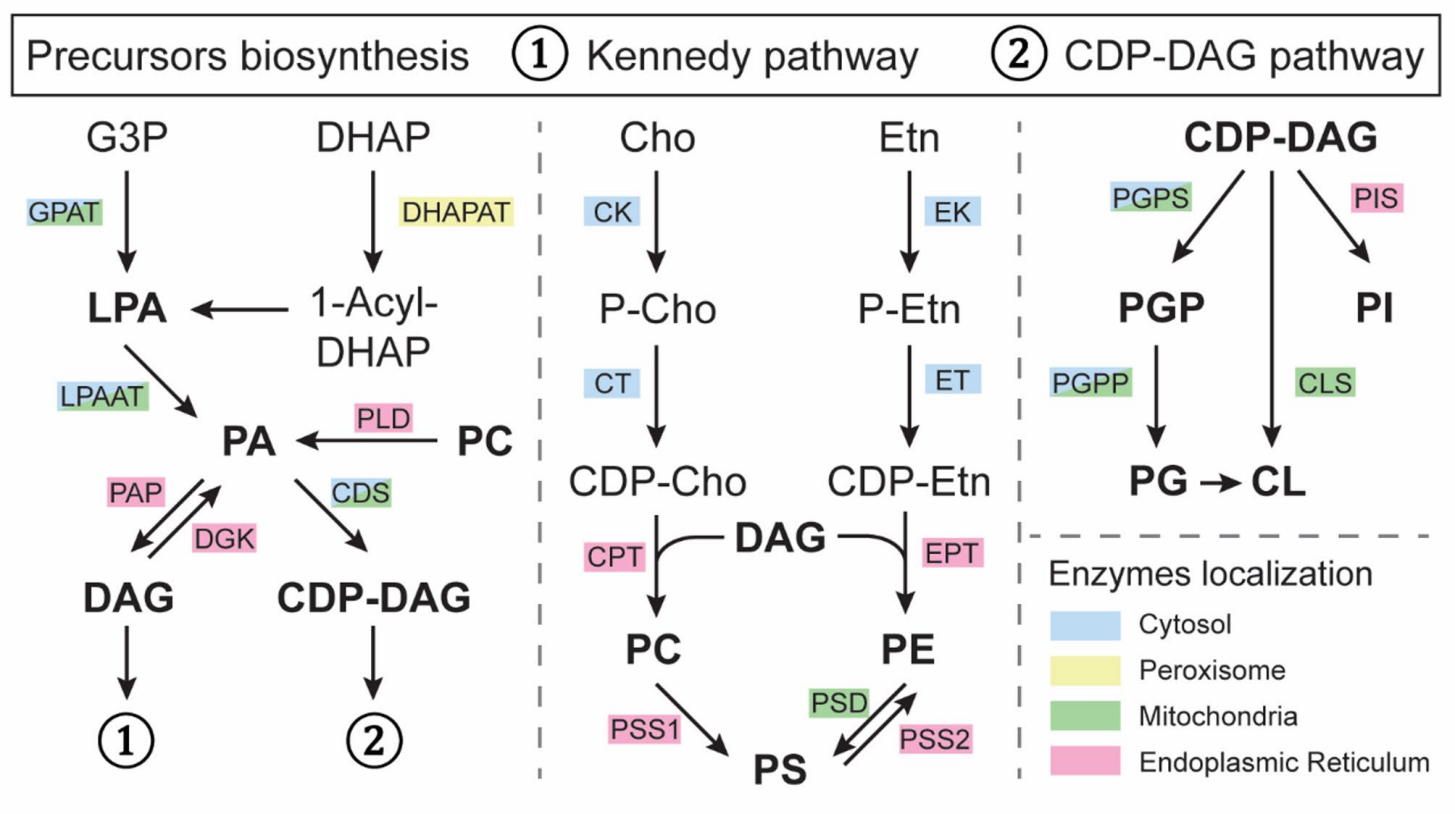
Biosynthesis of diacyl GPLs. Phosphatidic acid (PA) is the common precursor of GPLs, which produces either diacylglycerol (DAG) or cytidine diphosphate-DAG (CDP-DAG) (**1**). The Kennedy pathway takes DAG to form phosphatidylcholine (PC), phosphatidylethanolamine (PE), and phosphatidylserine (PS) (**2**). CDP-DAG is used to produce phosphatidylinositol (PI), phosphatidylglycerol (PG), and cardiolipin (CL). CDP-Cho/Etn cytidine: cytidine diphosphate-choline/ethanolamine, CDS: CDP-DAG synthase, Cho: choline, CK/EK: Cho/Etn kinase; CLS cardiolipin synthase; CPT/ETP: DAG Cho/Etn-phosphotransferase, CT/ET: Cho/Etn cytidylyltransferase, DGK: DAG kinase, DHAP: dihydroxyacetone phosphate, DHAPAT: DHAP acetyltransferase, Etn: ethanolamine, G3P: glycerol-3-phosphate, GPAT: G3P acetyltransferase, LPA: lysophosphatidic acid, LPAAT: LPA acetyltransferase, P-Cho/Etn: phosphorylated Cho/Etn, PAP: PA phosphatase, PGP: PG phosphate, PGPP: PG phosphate phosphatase, PGPS: PG phosphate synthase, PIS: PI synthase, PLD: phospholipase D, PSD: PS decarboxylase, PSS1/2: PS synthase 1/2

**Fig. 3:**
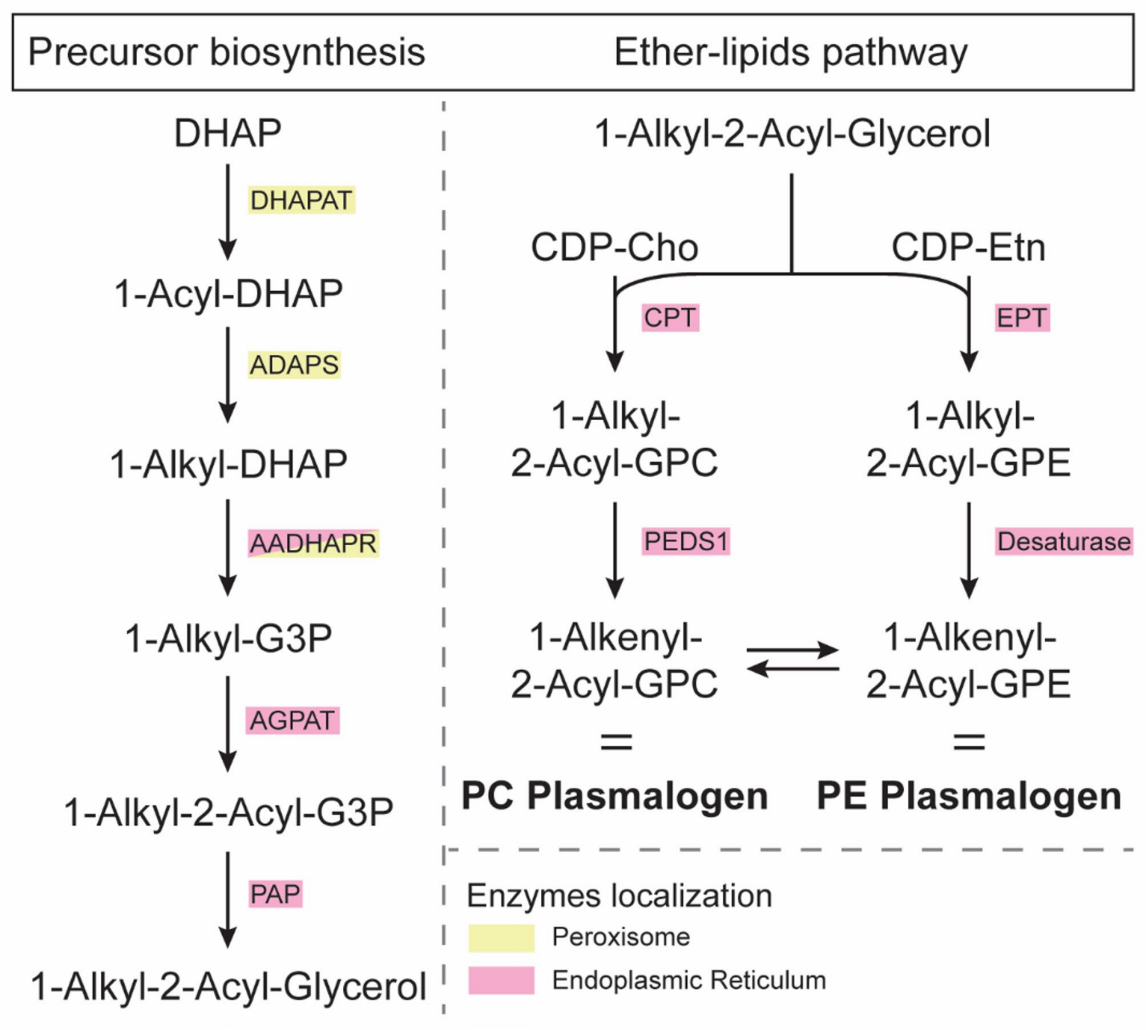
Biosynthesis of ether-linked GPLs. The diacylglycerol (DAG) analog 1-alkyl-2-acyl-glycerol is formed from dihydroxyacetone phosphate (DHAP) in peroxisomes and then in the reticulum endoplasmic. Then, 1-alkyl-2-acyl-glycerol is incorporated in the ether-lipids pathway, similarly to the Kennedy pathway, to form PC and PE plasmalogens. AADHAPR: alkyl/acyl DHAP reductase, ADAPS: alkyl DHAP synthase, AGPAT: alkyl G3P acetyltransferase, CPT/EPT: 1-alkyl, 2-acyl-glycerol Cho/Etn-phosphotransferase, DHAPAT: DHAP acetyltransferase, G3P: glycerol-3-phosphate, Cho: choline, Etn: ethanolamine, GPC/GPE: glycerol-3-phosphocholine/phosphoethanolamine, PAP: phosphate phosphohydrolase, PEDS1: plasmanylethanolamine desaturase 1

GPL synthesis predominantly takes place in the endoplasmic reticulum (ER), with contributions from mitochondria and peroxisomes [27, 28] (Figs. 2 and 3). GPL synthesis relies on the formation of phosphatidic acid (PA), a crucial precursor, that can be formed *via* three pathways [29] (Fig. 2). First, the acylation of glycerol-3-phosphate (G3P), by an acyl-CoA glycerol-3-phosphate acyltransferase (GPAT), or the acetylation of dihydroxyacetone phosphate (DHAP), can produce lyso-phosphatidic acid (LPA) that is converted to PA by adding a long-chain fatty acid through the action of the LPA acyltransferase (LPAAT) [30, 31]. Second, diacylglycerol (DAG) can be phosphorylated by the diacylglycerol kinase (DGK) to produce PA [32]. Third, phospholipase D (PLD) can hydrolyze existing GPLs to form PA [33]. Once formed, PA can be metabolized in two pathways. In the Kennedy pathway, PA is dephosphorylated to DAG by the phosphatidic acid phosphatase (PAP), and subsequently used to produce phosphatidylcholine (PC), phosphatidylethanolamine (PE), and phosphatidylserine (PS). Alternatively, in the CDP-DAG pathway, PA reacts with cytidine triphosphate (CTP) to form cytidine diphosphate diacylglycerol (CDP-DAG), a reaction catalyzed by the CDP-DAG synthase (CDS) and producing phosphatidylinositol (PI), phosphatidylglycerol (PG) and cardiolipin [28]. GPL homeostasis highly depends on the correct fluxes of these pathways [34].

GPLs constitute approximately 50–60% of total lipids in a typical mammalian cell [28] (Table 1). PC is the most abundant GPL, particularly present in the outer leaflet of the plasma membrane [35]. PE is the second most abundant GPL, especially enriched in the inner leaflet of plasma membranes and mitochondrial membranes [35]. PI is present in smaller amounts but is crucial in signaling pathways. PS is present at lower levels than PC and PE, but remains important, and is mainly confined to the inner leaflet of the plasma membrane [36]. PG and PA are minor components but are key intermediates in lipid biosynthesis and signaling [28]. While low in abundance, CL is enriched in inner mitochondrial membranes [37].

PC and PE are similarly produced in three enzymatic steps *via* the CDP-choline and CDP-ethanolamine pathways, respectively (Fig. 2). First, phosphorylation of choline and ethanolamine by choline/ethanolamine kinase (CK/EK) is happening [38, 39]. In the second step, CDP-choline and CDP-ethanolamine are formed from CTP by phosphocholine/phosphoethanolamine cytidylyltransferase (CT/ET). Third, PC or PE is produced by the transfer of DAG to CDP-choline/ethanolamine by choline/ethanolamine phosphotransferases (CPT/EPT) [40, 41]. Additionally, PC and PE are synthesized in specific ER domains in close contact with mitochondrial membranes, called mitochondrial-associated ER membranes (MAMs). PC can be locally formed by the repeated methylation of PE through the activity of an N-methyltransferase [42], while PE is formed by the decarboxylation of PS by the phosphatidylserine decarboxylase (PSD), after its transport into mitochondria [43]. PS is created by base exchange from PC or PE by a PS synthase 1/2 (PSS1/PSS2) at MAMs [36]. Mitochondria lack pathways for *de novo* phospholipid synthesis, making MAMs indispensable for supplying precursors like PS and PE.

In the ER, phosphatidylinositol synthase (PIS) combines CDP-DAG and inositol to form PI and its numerous phosphorylated derivatives [44]. PG synthesis starts in both ER and mitochondrial membranes, with G3P and CDP-DAG to form PG-phosphate (PGP), through the action of phosphatidylglycerol phosphate synthase (PGPS). Subsequently, PGP is dephosphorylated by the PGP phosphatase (PGPP) to produce PG [28]. Cardiolipin is exclusively formed in the inner mitochondrial membrane by the cardiolipin synthase (CLS), which combines PG with a second molecule of CDP-DAG [37].

Ether-linked GPLs form a unique subclass. Unlike previously described GPLs, these lipids contain an ether-linked fatty alcohol at the *sn*-1 position and a fatty acid at the *sn*-2 position (Fig. 1). Plasmalogens, the most abundant type, make up 15–20% of cell membrane GPLs and are characterized by a vinyl-ether double bond [45]. Most of the plasmalogen headgroups contain a choline or an ethanolamine. Their synthesis starts in peroxisomes and finishes in the ER [46, 47] (Fig. 3). First, acetylation of DHAP by the DHAP-acyltransferase (DHAPAT) forms 1-acyl DHAP [48], which is then modified by the alkyl-DHAP synthase (ADAPS). This enzyme replaces the acyl group with an alkyl chain to form 1-alkyl-DHAP [49]. At peroxisomes or ER membranes, reduction of 1-alkyl-DHAP by the acyl/alkyl DHAP reductase (AADHAPR) generates 1-alkyl-G3P, an analog of LPA [50]. ER-located enzymes catalyze the last steps of plasmalogen synthesis. The 1-alkyl-G3P is acylated by the acyl/alkyl-G3P-acyltransferase (AGPAT) and dephosphorylated by PAP to produce 1-alkyl-2-acyl-G3P, an analog of PA [51]. Similar to the Kennedy pathway, a phosphoethanolamine head group is added by ETP to form 1-alkyl-2-acyl-GPE [46]. Finally, plasmanylethanolamine desaturase (PEDS1) introduces an alkenyl bond, resulting in alkenyl-phosphatidylethanolamine (PE plasmalogen) [52]. Alkenyl-phosphatidylcholine (PC plasmalogen) is mostly formed by modifying PE plasmalogen head group by CPT and CDP-choline [53].

#### Transport and partitioning mechanisms

Transport mechanisms maintain local lipid homeostasis and prevent the accumulation of newly synthesized lipids [54]. Spontaneous diffusion of GPLs is minimal and extremely slow in the cytosol, due to their poor solubility. Therefore, GPL trafficking relies on non-diffusional mechanisms, which include vesicles, lipid-transport proteins (LTPs), and close contacts between membranes [28]. Vesicles, delineated by a lipid bilayer, carry non-specific bulk GPLs, which are transported from the ER to the Golgi, and can travel along the secretory pathway to reach different organelles [55, 56]. While vesicles move large amounts of GPLs, they are relatively slow and non-specific. As a consequence, vesicular trafficking is not an adequate mechanism for the fine-tuning of GPL homeostasis. In contrast, LTPs and membrane contact sites allow rapid and specific lipid transport. LTPs have hydrophobic pockets that bind specific lipids, protecting them from the aqueous environment as they shuttle between organelles and membranes [57]. Besides exchanging material through vesicles and LTPs, cellular compartments make specific contacts by apposing their membranes. The giant ER network creates contact sites with the Golgi apparatus, mitochondria, endosomes, peroxisomes, lipid droplets, and plasma membrane, which enables efficient GPL transport across the cell [58].

GPL partitioning refers to the non-random lateral distribution of lipid species within membranes. GPLs segregate into distinct domains based on their physicochemical properties, including saturation, headgroup identity, and interaction with lipids and proteins [59]. GPL partitioning into specific membrane domains is a key mechanism for organizing the plasma and organelle membranes and regulating their functions [60]. Notably, lipid rafts are small, dynamic, and ordered domains enriched in saturated GPLs, cholesterol, and sphingolipids. They serve as platforms for protein sorting, signal transduction, and membrane trafficking [60]. Raft domains arise from the phase separation between coexisting ordered and disordered phases. Attractive and repulsive lipid/lipid interactions seem to play a fundamental role in GPL partitioning. Nonetheless, the precise mechanism remains to be established. In addition, the relative distribution of GPLs between the plasma membrane, ER, mitochondria, and lysosomes plays a crucial role in maintaining cellular homeostasis. For instance, polyunsaturated PC species are more abundant in the axon compartment than in the cell body of neurons [61].

GPLs are asymmetrically distributed between membrane leaflets. PE and PS are typically enriched in the inner leaflet, while PC species are found predominantly in the outer leaflet [62]. This asymmetry is crucial for various cellular functions, including signaling and membrane stability [63]. Flippases and floppases are ATP-dependent enzymes that selectively transport specific GPLs (mainly PC, PE, and PS) to the cytoplasmic leaflet and the exoplasmic leaflet, respectively. Scramblases are ATP-independent proteins that facilitate the bidirectional and non-specific movement of GPLs between the two plasma membrane leaflets, with higher lipid fluxes [63]. Combining the opposite actions of slow-but-constitutive flippases and floppases with fast-but-regulated scramblases dynamically regulates membrane asymmetry and GPL distribution [63].

#### Homeostasis regulation

GPL homeostasis is regulated by a dynamic remodeling of the acyl chains of GPLs. This process ensures the production of thousands of distinct lipid species, their turnover, repair, and the release of lipid mediators [34]. A key remodeling pathway is the Lands cycle, which involves two steps [64]. First, phospholipase A (PLA) enzymes, PLA_1_ and PLA_2_, remove fatty acids from the *sn*-1 or *sn*-2 position of GPLs, respectively, producing lysophospholipids and free fatty acids [65]. PLA_1_ releases saturated fatty acids (SFAs) or monounsaturated fatty acids (MUFAs), and are known for their specific role in producing lysophospholipids bioactive mediators, such as lysophosphatidylserine (LPS) and lysophosphatidylinositol (LPI) [66]. PLA_2_ enzymes have a wider range of functions depending on their catalytic mechanisms, localization, and structural features [67]. By targeting GLPs containing polyunsaturated fatty acids (PUFAs), PLA_2_ enzymes produce second messengers and lipid mediators, including prostaglandins and leukotrienes [65]. Therefore, PLA_2_ enzymes are important regulators of inflammation, beyond their role in membrane remodeling. In the second step of the Lands cycle, lysophospholipid acyltransferases (LPLATs) re-incorporate new acyl chains into GPLs at the *sn*-1 or *sn*-2 positions. Each LPLAT enzyme has substrate specificity for certain headgroups and fatty acid, which allows cells to adapt membrane composition in response to different physiological needs [68].

GPL catabolism involves three other types of phospholipases that hydrolyze the different chemical bonds on GPL molecules. Phospholipase B (PLB) hydrolyses both *sn*-1 and *sn*-2 acyl esters on the glycerol moiety [65], while phospholipase C (PLC) hydrolyses the bond between the phosphate and glycerol backbone. PLC enzymes are key in signal transduction, especially targeting phosphatidylinositol 4,5-bisphosphate (PI [4, 5]P_2_) and phosphatidylinositol 4-phosphate (PIP_4_). When activated, PLC generates two second messengers: inositol 1,4,5-trisphosphate (IP_3_), which triggers Ca^2+^ release from the ER, and DAG, which activates signaling molecules such as protein kinase C [69, 70]. Phospholipase D (PLD) cleaves the bond between the phosphate and the head group to produce PA and a free head group [71].

The PC/PE ratio is a key indicator of GPL homeostasis and a biomarker of lipid disequilibrium. It refers to the molar ratio of PC to PE, the two major classes of GPLs found in cellular membranes. In mammalian cells, the PC/PE ratio is typically around 3, meaning that there is about three times more PC than PE in cell membrane. However, this number is tissue and cell-type-specific. The ratio is maintained within a narrow range, and its modification has been identified as a major factor in obesity and aberrant skeletal muscle functions [72]. Increased PC/PE ratio results in the inhibition of the ER Ca^2+^ importer sarco/endoplasmic reticulum-ATPase (SERCA), disrupted Ca^2+^ homeostasis, and triggers ER stress [73]. Conversely, a lower PC/PE ratio results in a decrease in membrane potential and loss of membrane integrity, which can initiate inflammation through the release of cellular contents, increased influx of extracellular components, including cytokines [74]. Mitochondria, and especially MAMs, are highly sensitive to modification of the molar PC/PE ratio. Even a modest increase or decrease can profoundly impair mitochondrial dynamics and functions, including fission/fusion, oxygen consumption, cellular ATP levels, and the rate of ATP production [72].

#### Physiological functions in the nervous system

GPLs are fundamental for the architecture and function of neuronal membranes, influencing their fluidity, permeability, charge and consequently regulating synaptic transmission [75]. GPLs play a key role in the synaptic cycles of exocytosis and endocytosis, modulating synaptic membranes and vesicle dynamics [76]. The extensive restructuring of synaptic membranes required for effective neuronal communication involves the rapid modifications in GPL molecular species. Therefore, enzymes responsible for GPL remodeling and catabolism are often concentrated in presynaptic compartments to facilitate these local adjustments. For instance, PLA_2_ catalyzes the conversion of PC to LPC which stimulates exocytosis by promoting membrane fusion through the positive curvature induced by LPC [77]. Additionally, PUFA-containing GPLs enhance membrane fluidity and reduce mechanical stress during fusion events [78, 79].

PA is a cone-shaped GPL that induces negative membrane curvature and facilitates presynaptic membrane fusion. In addition, PA interacts with key protein regulators of vesicle fusion, including small GTPases and syntaxin-1 A [33]. As a consequense, membranes enriched in PA exhibit a higher probability of vesicle fusion events. In neurons, enzymes responsible for PA synthesis, such as PLD and DGK, are concentrated at synaptic sites, emphasizing the role of PA in modulating synaptic transmission [76]. Astrocytes also contribute to the regulation of synaptic activity through the production of GPLs and their delivery to neurons, adding an extra layer of regulation. Notably, astrocyte-derived PA has been shown to influence neurite outgrowth and dendritic branching [80].

Negatively charged headgroups in PI and PS are involved in each stage of neuronal transmission [79]. Specific kinases phosphorylate the head group of PI to form phosphoinositide (PIP) species, which is essential during the different steps of the synaptic vesicle cycle [81]. These lipids recruit specific proteins at the presynaptic membrane, facilitating interactions with proteins responsible for synaptic vesicle priming or docking [82, 83]. PS is involved in exocytosis of synaptic vesicles mediated by the SNARE complex assembly, by promoting the binding of synaptobrevin-2 and syntaxin [84, 85], while also interacting with regulatory proteins such as Synaptotagmin-1, Munc13 and Rabphilin [81, 86]. In addition, PS is involved in neural circuit refinement by acting on synapse pruning. PS is normally located in the inner leaflet of plasma membranes, facing the cytosol. However, when PS is translocated to the cell surface by a phospholipid-scramblase, it acts as an “eat-me” signal for microglia inducing synapse elimination [87–89].

Beyond their structural roles in neuronal communication, GPLs serve as reservoirs for second messengers and bioactive mediators. Arachidonic acid (ARA) and docosahexaenoic acid (DHA), two major PUFAs enriched in the nervous system, are released from GPLs by the action of PLA_2_ at plasma membranes. These free PUFAs can directly bind their receptors or undergo enzymatic conversion to various bioactive mediators. Notably, some of these metabolites can cross the cell membrane and act in endocrine, paracrine, and autocrine signaling. DHA-derived mediators (e.g., resolvins and protectins) have potent anti-inflammatory properties [90, 91], while ARA-derived mediators (e.g., prostaglandins, leukotrienes and thromboxane) trigger the early pro-inflammatory response [92]. Besides, these PUFAs and their derivates are implicated in synaptic transmission through the modulation of the endocannabinoid system [93] and promote neuronal survival and neurogenesis [94, 95].

Plasmalogens are found in high concentrations in the brain, particularly within myelin sheaths. Characterized by specific alkenyl chains at the *sn*-1 position and enriched with ARA and DHA at the *sn*-2 position, plasmalogens confer unique properties to neural membranes [53]. PC and PE plasmalogens form more condensed lipid bilayers that facilitate rapid membrane fusion events, crucial for synaptic activity [96, 97]. In myelin membranes, PE plasmalogens are abundant and may increase the packing density and decrease the membrane fluidity in order to stabilize the myelin sheaths [98, 99]. Additionally, plasmalogens possess antioxidant properties due to their vinyl ether bond at the *sn*-1 position, effectively protecting membranes from oxidative damage [100–102].

#### Role in skeletal muscle physiology

GPLs are essential for the physiological functions of skeletal muscles, influencing muscle health, metabolism, and performance. The correct lipid homeostasis, especially the ratio between PC and PE, is critical for maintaining membrane integrity and functions, directly affecting muscle adaptations and responses to stimuli, including changes in diet and physical activities [103, 104]. Alterations in the PC/PE ratio or the concentration of PUFA-containing GPLs have been linked to defects in insulin sensitivity, impaired glucose handling, and subsequent disruption of the functional integrity of muscles [103, 105, 106].

Skeletal muscles exhibit distinct GPL compositions based on their metabolic properties. For example, glycolytic muscles like the gluteus and oxidative muscles such as the soleus demonstrate different GPL profiles that reflect their functional demands [107]. This composition is vital for energy metabolism, as GPLs influence mitochondrial function by modulating the outer and inner mitochondrial membrane composition and shape. Cardiolipin and PE are particularly important for creating the characteristic curvature of the inner mitochondrial membrane to form the cristae, which are necessary to provide a large surface area for efficient electron transport chain (ETC) activity [108].

Mitochondria are closely associated with the sarcoplasmic reticulum facilitating the coupling between energy production and muscle contraction. GPLs participate in Ca^2+^ homeostasis by maintaining the membrane integrity to prevent any Ca^2+^ leaks. Additionally, PE levels within the sarcoplasmic reticulum influence SERCA activity, a key regulator that facilitates Ca^2+^ reuptake during muscle relaxation [103, 109]. Alterations of PE levels can impair SERCA activity, leading to impaired Ca^2+^ handling and inefficient muscle contraction cycles [110].

The functions of GPLs in skeletal muscles have mainly been investigated in the context of human pathology. Mutations in genes associated with GPL metabolism frequently lead to myopathies, with notable examples including mutations in the *CHKB* gene, which encodes the first enzyme in PC biosynthesis (Fig. 2). Such mutations are linked to muscular dystrophy, a disorder characterized by muscle wasting, weakness, and hypotonia [111, 112]. Patients exhibit reduced PC production and increased catabolism, resulting in enlarged and dysfunctional mitochondria [113, 114]. Similarly, mutations in the *Tafazzin* gene cause Barth syndrome, resulting in decreased cardiolipin levels and abnormal mitochondrial cristae that further impair oxidative capacity in muscles [115].

Another example is the Sengers syndrome caused by mutations in the *AGK* gene encoding the mitochondrial enzyme acylglycerol kinase (AGK) [116, 117]. Patients suffer from cardiomyopathy, skeletal myopathy, cataracts, and lactic acidosis after physical exercise. AGK is involved in the production of PA and LPA (Fig. 2). GPL molecular remodeling and level modification have been observed in other myopathies without a direct genetic link. In the muscles of patients suffering from Duchenne muscular dystrophy, PC levels are increased [118], while in the mdx mouse model, PC and PE levels are dysregulated and contain fewer PUFAs [119]. Interestingly, PC species accumulate in degenerative muscular areas, with changes of their fatty acyl chain composition, suggesting altered membrane flexibility of muscle fibers [120].

Physiological GPL composition is essential for neuromuscular junction (NMJ) development, maintenance, and function. Enzymes remodeling GPLs, particularly inositol GPLs, are found at the motor endplate and control synaptic transmission [121]. The postsynaptic membrane at the NMJ is characterized by a cholesterol-rich phospholipid bilayer, which provides a unique, highly ordered lipid environment. This specialized membrane domain influences the structure and arrangement of acetylcholine receptors, promoting a configuration that supports rapid and efficient synaptic transmission [122, 123]. Ether-linked GPLs might play a role in the formation and maintenance of these membrane microdomains, also called lipid rafts [124]. Deficiencies in plasmalogen levels impair the clustering of acetylcholine receptors and impact synaptic development, reducing the frequency of spontaneous synaptic vesicle fusion events, ultimately affecting muscle strength and motor performance [124]. 

### GPL alterations in ALS patients and models

#### Blood circulation

Blood sample collection is a simple and minimally invasive procedure, making it suitable for routine diagnosis and longitudinal monitoring of disease. Recent advances have increased our understanding of the roles of blood lipids, with over 150 GPL species identified in human plasma [125].

Recent studies reported significant alterations of GPL levels in blood plasma or in the serum lipidome of ALS patients, mostly in sporadic cases. Notably, some studies identified increased levels of PC, PE, PI, LPC, and PC/PE plasmalogens species [20, 126–128]. Conversely, other research indicated decreased levels of PC, PE, PS, LPC, LPE, LPS, and PE plasmalogens species [126, 129–131]. Despite some contradictory findings, these studies consistently suggest that GPL metabolism alterations are prevalent among ALS patients and correlate with disease progression [128, 132].

A longitudinal study highlighted a progressive decrease in PE levels and its derivatives as ALS advanced, contrasting with normal aging, where these GPLs typically increase [128]. GPLs, particularly PC and PE, have been identified as critical metabolites that discriminate ALS patients from controls [20, 127, 130, 131]. Furthermore, GPL alterations could distinguish between ALS patients based on the site of onset (spinal, bulbar, or respiratory) and their progression rates [20]. Sol et al. further identified two PC species (PC44:8 and PC36:4) with a high discriminative capacity between normal and fast progressors, suggesting that GPLs may serve as potential biomarkers for ALS disease progression [20]. Interestingly, the profile of PC and PC plasmalogens evolved along disease progression [130, 132].

GPLs could aid in diagnosing ALS mimics such as primary lateral sclerosis (PLS), where only UMNs are affected. In a comparative study of blood lipidomes of ALS and PLS patients, significant GPL alterations were observed exclusively in ALS patients. Specifically, PE species containing oleic acid and long polyunsaturated chains were progressively increased in ALS patients compared to PLS patients [130]. In addition, Bjornevik et al. identified 275 individuals who developed ALS, in 5 prospective cohorts. Among 404 identified metabolites, 2 PC species were positively associated with ALS risk, and 1 PC species was negatively associated [133]. However, none of the associations remained significant after multiple comparison adjustments. This underscores the need for more pre-symptomatic blood lipidomic studies to understand GPL dysregulations prior to disease onset. Interestingly, a longitudinal blood metabolomic study in mutant *SOD1*^*G93A*^ mice showed that alterations of PC metabolism occurred before symptom onset [134]. Collectively, these findings position GPLs as potential diagnostic and prognostic biomarkers for ALS.

Nevertheless, interpreting blood lipidome results can be challenging due to various influencing factors such as diet, smoking, physical activity, sex, age, and body mass index (BMI) [125, 135, 136]. These variables can introduce biases into study design and patient-control matching. This challenge is exacerbated in ALS patients who have reduced physical activity, and significant dietary changes during disease progression. Furthermore, Goutman et al. raised concerns regarding fasting protocols prior to blood sample collection [129]. Many studies required overnight fasting or 12-hour fasts from ALS patients, which may not always be ethical or feasible given their health conditions. Overall, blood lipidomic analyses in ALS often lack association with clinical variables or have not been controlled for dietary habits, and future studies will need a better standardization.

#### Cerebrospinal fluid

It is important to recognize that the plasma lipidome may not accurately reflect central nervous system alterations. While the blood-brain barrier (BBB) permeability might be perturbed in ALS [137], its impact on GPL transport between the blood circulation and the nervous system is unknown. A recent lipidomic study showed that changes in brain lipid profiles were poorly reflected in the blood lipidome of healthy mice [138]. Therefore, the interpretation of blood lipidomic data with respect to neural alterations should be made with caution. GPL level variations could potentially reflect progressive muscle atrophy or systemic defects in energy metabolism. CSF collection is more invasive than blood sampling but provides a closer reflection of neural alterations due to its proximity to the nervous system. Although CSF lipid content is approximately 300-fold lower than plasma levels, it contains over 250 GPL species and is considered the most valuable source for biomarkers in neurological disorders [125, 139].

In the CSF, GPLs are primarily found in lipoprotein particles, which are crucial for trafficking and distributing lipids between cells and regions of the central nervous system. Lipoprotein particles promote neuronal homeostasis, synaptogenesis, neurite outgrowth, and injury repair [140]. While the role and potential alterations of lipoproteins in the CSF of ALS patients have not been investigated, few studies have nonetheless performed lipidomic analyses [20, 141, 142]. Interestingly, all studies reported the increased levels of PC species as potent discriminant lipids and pointed toward PC metabolism abnormalities. Blasco et al. developed a predictive model for clinical evolution based on CSF lipidomic data [141]. This model revealed the involvement of 7 PC species. For instance, lower levels of choline plasmalogens PC(37:3p) and PC(32:1p) are associated with a slower decline in BMI, and higher levels of PC(40:6p) are associated with increased survival [141].

Following damage to neuronal cells, lipids, especially PC, can be released in the CSF [143]. Remarkably, the specific PC species altered in the CSF of ALS patients were also dysregulated in the cerebral cortex of symptomatic mutant *SOD1*^*G93A*^ mice [134, 141]. These results indicated the potential pathophysiological relevance of the GPLs identified in the CSF. In parallel studies comparing CSF and plasma samples from the same individuals, Sol et al. observed that changes in the CSF lipidome were less prominent than those in plasma and lacked discriminatory power [20]. The absence of similar dysregulations between the CSF and plasma suggests either the absence of leakage from the CSF to the plasma or highly regulated lipid homeostasis within the CSF, even under ALS conditions.

Unlike blood lipidomics, interindividual and preanalytical variations in CSF lipidomics remain poorly defined, complicating accurate data interpretation. Therefore, further longitudinal studies are necessary to elucidate the mechanisms underlying alterations in the CSF lipidome associated with ALS.

#### The cerebral cortex and UMNs

The spinal cord and in vitro models of LMNs have been the preferential source of material to study lipid alterations in ALS, while the cerebral cortex containing the UMNs has received comparatively less attention. Recent evidence supports a potential primary role of the cerebral cortex in disease onset and progression [144–146]. UMNs and LMNs are interconnected, with their degenerative processes being somatotopically related and temporally ordered, potentially involving distinct pathological mechanisms [146, 147]. Therefore, a comprehensive examination of both regions is essential for understanding lipid alterations in ALS.

Investigations into the frontal cortex lipidome in sporadic ALS patients with TDP-43 proteinopathy revealed decreased levels of PC and ether-linked PE species [148]. Notably, ether-linked GPLs were also downregulated in other TDP-43 proteinopathies, such as sporadic FTLD-TDP and *C9ORF72*-FTLD [148]. While common lipidomic alterations were observed across the ALS-FTLD-TDP-43 spectrum, specific defects were noted in each disorder. In another study, two plasmalogen species were significantly decreased in the white matter of the motor cortex of sporadic ALS patients [149]. Similarly, the analysis of the motor cortex of mutant *SOD1*^*G93A*^ rats only revealed the downregulation of one PE plasmalogen in symptomatic animals [150]. Altogether, these studies suggest that GPLs are not influenced by the ALS pathology in the motor cortex.

This limited occurrence of lipid alterations may result from a dilution effect, as UMNs constitute a relatively small subset among the numerous cell types in the cerebral cortex. GPL alterations might be confined to UMNs and invisible when examining the entire cerebral cortex. Indeed, transcriptomic analysis of purified adult UMNs from mutant *Sod1*^*G86R*^ mice unraveled cell-type-specific GPL alterations. Gene set enrichment analysis revealed sub-networks related to GPL biosynthesis, phosphoinositide metabolic process, and membrane lipid metabolic process [147]. This study indicates that UMNs harbor distinct transcriptomic changes, which could translate into altered GPL concentrations at metabolite level.

A recent study investigated the lipidome of iPSC-derived cortical neurons carrying C9 repeats and their isogenic controls. It showed that the presence of C9 repeats, rather than C9orf72 loss-of-function, drove a distinct shift toward increased GPL saturation and a reduction in highly polyunsaturated GPLs compared to controls [151]. These findings underscore the role of lipid dysregulation in cortical neurons, although this effect may be specific to C9-associated FTD.

#### The spinal cord and LMNs

In the spinal cord, early transcriptomic and lipidomic alterations of GPL metabolism have been described in rodent models. A meta-analysis of RNA-sequencing data from mutant *SOD1*^*G93A*^ mice indicated transcriptional dysregulation of GPL metabolic pathways at pre-symptomatic stages, which persisted at symptomatic stages. Notably, the PE biosynthetic pathway was upregulated [152]. At the metabolite level, most GPL species affected in the spinal cord of mutant *Sod1*^*G86R*^ models decreased before any electromyographic signs of NMJ denervation and motor impairments. PC, PE, and their plasmalogen forms were particularly affected, with dysregulations continuing at symptomatic stages [153, 154]. Imaging mass spectrometry technique has also shown reduced PC levels in the anterior horn of the spinal cord of end-stage mutant *SOD1*^*G93A*^ animals, suggesting a correlation between PC alterations and neuronal loss [155]. Interestingly, increased levels of ether-linked GPLs and plasmalogens were found in nuclei from the anterior horn of lumbar spinal cords of ALS patients, and confirmed in mutant *SOD1*^*G93A*^ mice and human MNs, highlighting alterations of the nuclear envelope that may relate to nucleocytoplasmic defects [156].

Mutations in the *SOD1* gene account for a small proportion of ALS patients (approximately 2%) and lead to a specific clinical presentation (mostly limb-onset, predominance of LMN involvement) [1]. Hence, the development of additional models that reflect the broader range of ALS phenotypes and their underlying disease mechanisms is crucial. Recently, untargeted lipidomic approaches applied to the spinal cord of *FUS* and *TDP-43* mouse models revealed significant differences [157, 158]. In *hFUS*^*+/+*^ mice expressing human wild-type *FUS*, we recently showed that symptomatic animals recapitulate the lipidomic pathological signature previously established in mutant *SOD1* models and in ALS patients. Notably, *hFUS*^*+/+*^ mice displayed increased LPC concentrations, rearrangements of PC and PE species, plasmalogen alterations, and decreased cardiolipin levels [157, 159]. Conversely, symptomatic *TDP-43*^*Q331K*^ mice revealed increased GPL concentrations and the extensive lipid metabolism restructuring. PC was the most significantly affected class among PE, PI, PS, PG, and LPC [158]. These different studies highlight common GPL dysregulations between mouse models with diverse genetic backgrounds and disease phenotypes.

In vitro models are valuable tools for dissecting the cellular and molecular mechanisms leading to neurodegeneration. For instance, Lanznaster et al. developed a fast and reproducible TDP-43 toxicity model using a human-derived cell line (HEK293T) overexpressing TDP-43. This model recapitulated the decreased levels of PC and increased levels of LPC observed in previously described models [160, 161]. Recently, Lee et al. analyzed the selective vulnerability of spinal LMNs and resistance to neurodegeneration of ocular LMNs. By using a multi-omics approach on 17 human induced pluripotent stem cells (iPSCs) cell lines, including *SOD1*^*A4V*^, *C9ORF72*, *TPD-43*^*Q343R*^, and sporadic ALS patient cell lines, the authors identified common defects in GPL metabolism in spinal MNs. GPL metabolism was significantly upregulated at RNA and metabolite levels [162]. This study demonstrated that GPL alterations could be involved in a common pathologic pathway in sporadic and familial ALS and that this could contribute to spinal MN vulnerability.

#### Skeletal muscles and NMJs

Recent evidence demonstrated that skeletal muscles play an active role in ALS pathology from early disease stages, with changes occurring independently of MN loss and NMJ denervation, in a muscle cell-autonomous manner [163, 164]. Alterations in muscle lipid metabolism have been described in ALS patients and several mouse models, including mutant *SOD1*^*G93A*^, *Sod1*^*G86R*^, and *TDP-43*^*Q331K*^ mice [154, 158, 165]. These changes are characterized by an early shift in mitochondrial fuel preference from glucose to lipid and an elevated lipolysis rate during disease progression [165–167]. Despite the major role of lipids in muscle functions, only two studies explored the lipidomic pathological signature in skeletal muscles in ALS models [154, 158]. In the soleus (oxidative muscle) of pre-symptomatic and symptomatic mutant *Sod1*^*G86R*^ mice, and in the tibialis anterior (predominantly glycolytic muscle) of symptomatic *TDP-43*^*Q331K*^ mice, increased levels of various GPL species were observed, including PC, PE, PI, PG, PS, LPC [154, 158]. Similar alterations in PC and LPC species were confirmed in the triceps surae (mix of oxidative and glycolytic muscle) of symptomatic mutant *SOD1*^*G93A*^ mice using a targeted approach focused on PC metabolism.

Interestingly, these results indicate common alterations independently of the metabolic status of the different skeletal muscles. During the progression of ALS, skeletal muscles are differentially affected depending on their metabolism. Glycolytic muscles are more severely affected than oxidative muscles, while muscle fibers progressively shift from a glycolytic to an oxidative metabolism [166, 168, 169]. Therefore, untargeted lipidomic analyses are required to understand how these metabolic changes affect GPL composition in different skeletal muscles of ALS patients and models.

GPLs may play multiple roles in ALS muscle pathology. Studies in Duchenne muscular dystrophy mouse models suggest that changes in PUFA-containing GPL levels might be involved in NMJ dysfunction and altered morphology (fragmented endplates, size, shape) [170–172]. Interestingly, ALS patients and models share common alterations of PC metabolism with patients and models of Duchenne muscular dystrophy [173]. As ALS NMJs are also characterized by similar altered morphology [163], one can hypothesize that muscle-specific PC species also participate in NMJ pathology in ALS, in accordance with the dying-back hypothesis. Lipid membrane alterations at the motor endplate occur in the pre-symptomatic stage in mutant *SOD1*^*G931A*^ mice, correlating with increased extracellular choline levels and NMJ fragmentation. These early abnormalities were accompanied by a decrease in lipid raft markers and an increase in membrane fluidity [174]. Data in mutant *SOD1*^*G931A*^ and ∆FUS(1-359) mice suggest that ceramide accumulation is the main driver of lipid raft disturbances [174, 175]. However, the implication of GPLs, and particularly plasmalogens, should not be excluded. Interestingly, overexpression of a lipid raft organizer protein, caveolin, had beneficial effects on motor functions and survival in mutant *SOD1*^*G931A*^ mice [176, 177].

A recent study demonstrated the involvement of LPA as a deleterious pro-inflammatory mediator in the skeletal muscles of mutant *SOD1*^*G93A*^ mice [178]. Deleting its receptor LPA_2_ using a knock-out model delayed disease onset, reduced motor decline and muscular atrophy, but unexpectedly decreased the lifespan. Investigation of the muscle pathology demonstrated that LPA_2_ signaling triggers inflammation and promotes macrophage invasion into skeletal muscles, contributing to muscle atrophy [178]. Thus, the role of GPLs in muscle pathology might be plural and will require more in-depth studies.

### Key contributing factors to disease mechanisms

#### MAMs: a critical pathogenic hub in ALS

MAMs are specialized regions where ER and mitochondrial membranes are closely apposed, facilitating communication and the transfer of lipids, Ca^2+^, and other signaling molecules. MAMs are crucial homeostatic hubs regulating Ca^2+^ metabolism, autophagy, and lipid metabolism [42]. These cellular pathways were extensively associated with ALS pathology, and several ALS-causative genes identified are involved in processes that maintain MAM integrity and function, highlighting the central role of MAM dysfunction in the development of ALS [6].

Mutant SOD1, FUS, and TDP-43 proteins have been found accumulating in aberrant mitochondrial compartments, disrupting MAM function [179–181]. Their mislocalization and gain-of-toxic function effects reduced the number of ER-mitochondria contact sites and impaired the interaction between VAPB and PTPIP51, key proteins involved in MAM tethering [182]. Mutant FUS, TDP-43, and neurotoxic dipeptide repeat proteins generated from C9ORF72 expansions modulated GSK3β kinase activity, specifically by promoting phosphorylation at serine 9. This modification led to the dissociation of VAPB and PTPIP51, thereby impairing Ca^2+^ exchange between the two organelles [179, 180, 183]. Additionally, mutations in VAPB have been associated with ALS [184]. These mutations increased the interaction of VAPB with PTPIP51, resulting in Ca^2+^ release from the ER, which can be harmful by either raising Ca^2+^ levels in mitochondria or disrupting mitochondrial transport [185]. SIGMAR1 is another protein genetically implicated in ALS that is enriched at MAMs with an abnormal distribution found in SOD1-ALS and VAPB-ALS patients, as well as sporadic patients [186, 187]. In neurons, loss of SIGMAR1 reduced MAM formation through weakened interaction with IP3R3. Disruption of this interaction impaired Ca²⁺ signaling, disturbed mitochondrial dynamics, and contributed to ER stress [188]. Additional MAM-associated proteins, such as VDAC1, TBK1, MFN1/2, and DRP1, have been linked to ALS pathogenesis, further underscoring the critical role of MAMs in maintaining neuronal health [189].

GPL composition in MAMs is critically important for maintaining their functions and integrity. GPLs influence membrane properties and contribute to the formation of lipid raft microdomains that anchor key proteins like SIGMAR1 and IP3R3, thereby facilitating Ca^2+^ signaling and stabilizing MAMs [189]. Lipidomic analyses demonstrated specific alterations in GPL composition in MAM extracts from both mice and human tissues [181, 190]. Notably, in fibroblasts and brain extracts from sporadic ALS patients, as well as in the brains and spinal cord of mutant *SOD1*^*G93A*^ mice, PC and PE species were consistently altered. Interestingly, PC species displayed opposite patterns of alterations in the brain *versus* the spinal cord [181]. Additionally, levels of PS species were altered, with an increase of species containing SFAs [181]. These findings suggest that disrupted GPL composition in MAMs may contribute to the impairment of their functions in ALS.

A recent study demonstrated the pivotal role of MAM disruption in the cascade of pathological events in ALS. Especially in models with SOD1 mutations, MAM dysfunction impaired glucose-derived pyruvate metabolism, forcing cells to rely on fatty acid oxidation. This metabolic shift led to mitochondrial stress, increased reactive oxygen species production, and ultimately, bioenergetic defects, a hallmark of ALS MN pathology [181]. Interestingly, enhancing the expression of the MAM tethering protein MFN2 restored mitochondrial alterations in ALS models carrying TDP-43 mutations [191]. MAM disruption could also affect other pathological mechanisms linked to ALS. For instance, MAMs influence autophagosome formation by modulating GPL availability and serve as a critical source of lipids for correct autophagic flux [192]. Both PE and PC are critical for autophagosome membrane expansion. PE binds LC3-II, promoting phagophore closure [193]. MAM disruption altered ATG14 localization and suppressed autophagosome formation [194]. Since autophagic and lysosomal functions are impaired in ALS [195], further research should certainly evaluate the contribution of MAM and GPL alterations.

Disrupted MAMs correlate with GPL alterations at symptomatic stages in the spinal cord of *hFUS*^*+/+*^ mice [157, 179]. In vitro, our research group identified a reduced number of MAMs correlating with reduced PC levels using iPSCs-derived spinal MNs and oligodendrocyte progenitor cells (OPCs) from ALS patients harboring FUS mutations (P525L and R521H) [196, 197]. In OPCs, MAM dysfunction is also correlated with ER stress, Ca^2+^ signaling defects, and altered mitochondrial respiration [197]. These studies demonstrated common GPL alterations in FUS pathology. In addition, astrocyte primary cultures from mutant *SOD1*^*G93A*^ mice also showed decreased levels of PC when co-cultured with MNs upon glutamate stimulation [198], supporting the idea that PC alterations and potentially also MAM defects are not strictly limited to MNs.

#### Impaired glial communication and functions

Beyond neuronal cell-intrinsic alterations, it is now well-established that glial cells actively participate in MN degeneration through gain-of-toxicity and loss-of-support mechanisms [199]. Astrocytic processes physically interact with synapses, forming the “tripartite synapse”, allowing for the local transfer of lipids. Notably, PA is transferred within the synaptic region and impacts neurite outgrowth, synaptic physiology, and neurotransmission [80, 200]. Interestingly, PA was decreased in the spinal cord of symptomatic *hFUS*^*+/+*^ mice [157]. Conversely, extracellular LPA, possibly secreted by neurons, can induce astrocyte proliferation and the secretion of EGF ligands, leading to axonal growth [201, 202]. Recently, it was demonstrated that astrocytes can cause neuronal death via the secretion of saturated lipids contained in APOE and APOJ lipoparticles [203], highlighting the critical role of astrocytes as mediators of bioactive lipids in the central nervous system. Modification of membrane GPL composition in astrocytes may also exacerbate neuroinflammation. During astrocyte activation, oxidative stress and inflammatory signals upregulate PLA_2_ expression, triggering ARA release from membrane GPLs [204]. *Postmortem* studies in the spinal cord of ALS patients indicated that PLA_2_ expression is upregulated in reactive astrocytes, further contributing to the vicious circle leading to MN degeneration [205]. In addition, we recently conducted a lipidomic analysis of human iPSC-derived astrocytes harboring the FUS R521H mutation. Data showed a reduction in PC and PI accompanied by discret alterations in PE and plasmalogens [206]. The functional impact of these alterations still needs to be investigated.

Microglial function is tightly regulated by various GPLs influencing neuroinflammation, phagocytosis, and neuronal health. PC synthesis is essential for inflammatory cytokine secretion and acts as a ligand for TREM2. Furthermore, remodeling of specific PC species, like arachidonic acid-containing PC, contributes to microglial activation after injury [207, 208]. LPC, derived from PC breakdown *via* PLA_2_, triggers morphological changes (de-ramification) and IL-1β release in activated microglia through distinct pathways [209]. Interestingly, LPC was increased in the spinal cord of ALS patients as well as ALS models and linked to MN degeneration [153, 157, 210, 211]. In addition, LPA also drives microglia towards a pro-inflammatory phenotype *via* the MAPK-dependent pathways [212]. In contrast, extracellular cardiolipin enhances microglial phagocytosis and neurotrophic support while reducing inflammation, suggesting a neuroprotective role in neurodegenerative conditions [213, 214]. In ALS, cardiolipin levels are decreased in the spinal cord of mutant *SOD1*^*G93A*^ mice and *hFUS*^*+/+*^ mice [150, 157, 215], which could suggest a decreased neuroprotective role. Further research is needed to elucidate the role of GPLs specifically on microglia and their impact on MN survival.

Through myelination, oligodendrocytes provide metabolic support to neurons and insulate the axons. The predominant GPLs in myelin are PC, PE, and PE plasmalogens, which are critical for maintaining myelin integrity, stability, and function [216]. The long-chain of SFAs and MUFAs within these GPLs contributes to the electrical insulation of the membrane of the axons by increasing its order and rigidity, thereby promoting tight lipid packing within the bilayer [216]. Beyond structural roles, plasmalogens also function as reservoirs for inflammatory signaling molecules and are involved in modulating oxidative stress and cell death pathways [217, 218]. Increasing evidence suggests that significant alterations occur in the myelin sheath, both structurally and chemically [219]. Regarding GPLs, only subtle changes in plasmalogen species were detected in the cortical white matter of ALS patients [149]. However, in vitro studies showed altered GPL metabolism in human OPCs carrying FUS mutations, notably PC and PI species [197]. How these lipid dysregulations contribute to the white matter degeneration observed in ALS remains to be fully understood.

#### Altered membrane biophysical properties

Membrane biophysical properties are regulated by constant deacylation and reacylation reactions mediated by enzymes of the Lands cycle [64]. Phospholipases and acyltransferases are highly active in the brain and spinal cord, and alterations in the Lands cycle have been linked to disruptions of the GPL homeostasis and neurodegeneration [140]. In both mutant *SOD1* mouse models and ALS patients, increased levels of phospholipase mRNA and protein were observed [153, 157, 205, 210, 220]. Notably, PLA_2_ activity was almost four-fold increased in the grey matter of spinal cords of ALS patients [205, 210]. PLA_2_ abnormal activity enhanced GPL species degradation and LPC accumulation in the spinal cord of ALS patients and mouse models [153, 154, 211]. This accumulation could contribute to the ALS pathology as LPC has potent demyelinating and inflammatory properties and was toxic for human spinal MNs in vitro [210, 221, 222]. A recent study in mutant *SOD1*^*G93A*^ mice showed that the accumulation of misfolded mutant SOD1 in MNs triggered PLA_2_ upregulation *via* TNFα induction [223]. Furthermore, inflammatory molecules generated from lipids released by phospholipase activity, such as prostaglandin E2, were elevated in the serum and CSF of ALS patients [224].

Alteration of GPL remodeling enzymes could impact GPL saturation levels and thereby membrane properties. Increased unsaturation results in an increase in membrane fluidity. Hence, PUFAs increase membrane fluidity, while SFAs and MUFAs increase membrane stiffness. Only a few studies investigated the membrane fluidity properties in ALS, leading to contradictory results. The analysis of the lipid composition in lipid rafts extracted from the spinal cord of sporadic ALS patients pointed towards an increased membrane fluidity as a result of differences in fatty acid profiles [225]. On the contrary, Miana-Mena et al. reported that in mutant *SOD1*^*G93A*^ mice, membrane fluidity was reduced using fluorescence spectroscopy [226]. This might be due to increased PUFA peroxidation in membrane phospholipids in response to mutant SOD1-induced oxidative stress rather than remodeling of the membrane PUFAs [226]. A recent study explored membrane fluidity in a more physiological paradigm by using the ability of flies to recover from cold stress. *Drosophila* change their feeding preferences in response to cold exposure to incorporate more PUFAs into their lipid bilayers to maintain their membrane fluidity [151]. After cold exposure, all control flies showed a full recovery, while only 2% of C9 flies expressing (G4C2) 36 exhibited a full recovery [151]. These data suggest a detrimental decrease in membrane fluidity in the context of C9ORF72 repeat expansions. Altogether, these contradictory results highlight different potential pathways leading to alterations in membrane properties during ALS progression depending on the genetic background and on the underlying pathophysiological mechanisms.

#### Importance of the PC/PE ratio

The PC/PE ratio in skeletal muscle plays a significant role in metabolic health and cellular functions. Studies in humans and mice demonstrated that unbalanced synthesis of muscle PC and PE influences muscle insulin sensitivity by disrupting cellular Ca^2+^ homeostasis through reduced SERCA activity [109, 227]. Analysis of sarcoplasmic reticulum-enriched fractions revealed decreased GPL content in individuals with metabolic syndrome compared to controls, suggesting a potential mechanism underlying impaired insulin-stimulated glucose disposal [228]. Since ALS patients frequently develop insulin resistance [19], further research should evaluate how the progressive alterations of GPL levels in skeletal muscles could potentially contribute to metabolic alterations, or vice versa.

Our understanding of this ratio within the brain remains limited. Each brain region exhibits a distinct lipid molecular signature, and lipid profiles are further influenced by specific cell types, contributing to significant heterogeneity [17, 229]. This complexity makes it challenging to generalize findings or compare results across studies. Moreover, the PC/PE ratio can fluctuate rapidly in response to disease or metabolic changes, with alterations that may be transient [28]. In a FUS-ALS context, we previously observed that the PC/PE slightly decreased in the spinal cord of symptomatic *hFUS*^*+/+*^ mice and in OPCs in vitro, primarily driven by a decrease in PC concentrations [157, 197]. Further research is necessary to elucidate the potential role of the PC/PE in the nervous system.

Alterations in the expression or activity of enzymes involved in the Kennedy pathway, which controls the synthesis of both PC and PE, have a direct impact on the cellular PC/PE ratio. The metabolic interconnection of PC, PE, and PS biosynthesis is further regulated by MAM-resident enzymes [28]. Specifically, PS is synthesized from PC and PE by the enzymes PSS1 and PSS2, respectively, while in mitochondria, PS is decarboxylated by PSD to yield PE. This tight coupling ensures a dynamic balance among these GPLs [28]. How alterations in these enzymes that regulate lipid biosynthesis, and impact the PC/PE ratio, might contribute to ALS pathology remains to be studied in detail.

#### Plasmalogens modulate oxidative stress and neuroinflammation

Plasmalogens, a class of ether-linked GPL, with antioxidant and anti-inflammatory properties, are increasingly recognized for their role in neurodegeneration [53, 148]. In ALS patients, plasma plasmalogens levels were associated with poorer survival and showed a negative correlation with the ALSFRS score [230, 231]. Functionally, plasmalogens terminate lipid oxidation and protect ROS-vulnerable membranes, especially myelin, acting as endogenous antioxidants. Elevated oxidative stress in ALS contributes to neuronal damage, and plasmalogen deficiency may exacerbate this process. Plasmalogens are synthesized primarily in peroxisomes [46], and ALS has been linked to peroxisomal disturbance, including reduced expression of AGPS, a critical enzyme in plasmalogen synthesis (Fig. 3) [148, 156]. Impaired peroxisomal function may thus directly contribute to plasmalogen depletion in ALS and could further exacerbate oxidative stress. Additionally, plasmalogen depletion may worsen neuroinflammation by modulating lipid raft distribution of membrane proteins and activating G protein-coupled receptors, leading to reduced microglial activation and lower production of pro-inflammatory cytokines [232]. Conversely, ongoing inflammation and oxidative stress could further deplete plasmalogen levels, creating a vicious cycle [46]. However, studies on ALS patients and mouse models reported both decreased and increased plasmalogen levels in ALS, making their precise contribution to antioxidant defense and neuroinflammation unclear.

### Emergent biomarkers and novel therapeutic strategies

The ALS community urgently needs potent biomarkers to mitigate the significant diagnostic delay and to accurately evaluate disease progression rates. The integration of valid biomarkers in clinical practice can considerably enhance patient follow-up in clinical trials, and potent early-stage biomarkers could lead to shorter clinical trials with smaller cohorts [233]. Recent research has identified lipidomics as a promising avenue for discovering new biomarkers [234], primarily focusing on blood and CSF samples from sporadic ALS patients. Notably, PC species emerged as promising diagnosis and prognosis biomarkers of ALS, with PC(36:4) identified as a significant discriminant feature in both blood and CSF [20, 131, 141].

Despite these advancements, many proposed lipid biomarkers, including GPLs, lack validation in large-scale studies and are not yet utilized in clinical settings. This gap is largely attributed to their low specificity and inability to distinguish ALS from disease mimics. As a consequense, the reproducibility and validation of new lipid biomarkers remain critical challenges. Moreover, it is uncertain whether alterations in GPL levels in blood and CSF genuinely reflect the underlying pathological processes or merely represent lifestyle changes among ALS patients, such as dietary and physical activity modifications that influence the blood lipidome. Hence, it remains very challenging to establish a clear link between GPL modifications and the progressive neuronal damage occurring in ALS patients.

In recent decades, phospholipases were recognized as significant contributors to neurological disorders, including ALS [150, 153, 157, 205, 210, 223]. Increased activity of these enzymes may trigger oxidative stress and inflammation within the nervous system. A genome-wide association study in ALS patients uncovered PLC delta 1 (PLCD1) as a potential disease modifier [235]. Interestingly, PLCD1 genetic ablation showed a protective effect in mutant *SOD1*^*G93A*^ mice, delaying the onset of motor symptoms and increasing survival [235]. At the cellular level, PLCD1 removal prevented spinal MN nuclear shrinkage but did not affect gliosis [235]. Recently, a genome-wide screen for ALS modifiers using *Drosophila* carrying *FUS* or *TDP-43* mutations revealed PLD as a potent ALS modifier [236]. However, the genetic deletion of the enzyme has only transient beneficial effects regarding functional motor deficits, without impacting survival [236]. In mutant *SOD1*^*G93A*^ mice, pre-symptomatic reduction of cytosolic PLA_2_-α expression with ASOs beneficially impacted neuroinflammation and glial activation and further delayed motor decline with a modest increase in survival [220]. Overall, studies targeting phospholipases in ALS showed limited efficacy. While several phospholipase inhibitors were reported, their mechanisms of action, specificity, selectivity, and potential side effects remain poorly understood, hindering their translation to clinical applications for other neurological disorders.

Mitochondrial dysfunction is central to various ALS pathological mechanisms and may serve as a pivotal factor leading to MN degeneration. Mutated proteins associated with ALS, such as SOD1, TPD-43, FUS, and C9ORF72, interact with mitochondria and accumulate within the organelle, causing defects in respiration and ATP production [237]. Disruption of ER-mitochondria communication contributes to Ca^2+^ homeostasis perturbations, aberrant fission/fusion dynamics, and dysfunctional autophagy/mitophagy [179, 180, 191, 238, 239]. Cardiolipin, a unique GPL found exclusively in mitochondria, plays a crucial role in several aforementioned mechanisms [240]. Its levels are notably decreased in the spinal cord of *SOD1* and *FUS* rodent models [150, 157, 215]. Liu et al. explored the role of the acyl-CoA: lysocardiolipin acyltransferase 1 (ALCAT1), the key enzyme in cardiolipin remodeling that catalyzes acylation of lysocardiolipin [215, 241]. ALCAT1 expression was abnormally elevated in the skeletal muscles of mutant *SOD1*^*G93A*^ mice [215]. Treatment with a pharmaceutical inhibitor of ALCAT1 (Dafaglitapin) demonstrated beneficial effects on LMN dysfunction, ROS levels, neuronal inflammation, and muscle atrophy. It also delayed disease onset and moderately extended lifespan [215]. Gautman et al. developed a different in vivo approach using SBT-272, a well-tolerated small molecule crossing the BBB, that stabilizes cardiolipin within mitochondrial inner membranes [242]. SBT-272 treatment demonstrated a neuroprotective effect on UMNs in the motor cortex of mutant *TDP-43*^*A315T*^ mice. The therapy improved the ultrastructural integrity of mitochondria, increased UMNs arborization and branching, and improved the survival of mice [242].

MAM activity is involved in multiple pathological mechanisms disrupted in ALS, highlighting its potential as a critical hub for neurodegeneration and disease progression [189]. Thereby, targeting MAMs appears as a relevant therapeutic approach for ALS. In this context, recent studies showed that improving the formation and activity of MAM domains can rescue mitochondrial metabolic alterations and lipid metabolism defects [181, 243]. A recent high-throughput screening, designed to find small molecules capable of improving MAM functions and number, provided further evidence that targeting these structures could be a promising strategy for treating ALS [243]. One promising modulator demonstrated its ability to shorten the distance between mitochondria and ER membranes in cellular models and restore bioenergetic parameters, cristae structure, and lipid alterations in lymphoblasts from sporadic ALS patients [243]. These data suggest that MAM disruption acts upstream of mitochondrial and lipid alterations in ALS.

Using a genome-wide screening approach in Drosophila we recently identified Glycogen Synthase Kinase 3 beta (GSK3β) as a novel modifier of FUS-ALS pathology [244]. As previously described, FUS pathology leads to GSK3β hyperactivation, disrupting MAMs and the mitochondrial ultrastructure [180, 244]. Interestingly, pharmacological inhibition of GSK3β restored MAM numbers and normalized mitochondrial Ca^2+^ levels in FUS-expressing NSC34 cells [180]. Additionally, we demonstrated that genetic and pharmacological inhibition of *shaggy*, the *Drosophila* ortholog of GSK3β, rescued the motor phenotype and increased the lifespan of FUS flies [244]. Several studies established a connection between MAM integrity, GPL alterations, and FUS pathology [157, 159, 196, 197]. In MNs and OPCs derived from FUS-ALS patient iPSCs, we found that MAM integrity correlated with decreased PC levels and transcriptomic alterations related to GPL metabolism [196, 197].

In a therapeutic approach targeting the histone deacetylase 6 (HDAC6) using antisense oligonucleotides or with pharmacological inhibitors (Tubastatin A and ACY-738), we demonstrated the successful rescue of MAM integrity and PC levels in MNs derived from FUS-ALS patient iPSCs [196]. Additionally, we treated *hFUS*^*+/+*^ mice with the potent HDAC inhibitor ACY-738, which substantially extended the lifespan of the animals, reduced motor decline, weight loss, and muscular atrophy [159]. These beneficial effects on FUS pathology correlated with the rescued GPL alterations in the spinal cord [157, 159]. Nevertheless, the effect of ACY-738 treatment on MAM integrity remains to be confirmed in vivo. How HDAC inhibitors restore MAM integrity and target GPL metabolism is still unclear. The ACY-738 treatment demonstrated a multifaceted effect on *hFUS*^*+/+*^ mice, inducing histone hyperacetylation, which led to chromatin relaxation and increased accessibility. As a result, there was a significant restoration of gene and protein expression patterns linked to lipid metabolism that was dysregulated in the ALS mouse model [159]. Hence, the normalization of GPL levels may be attributed to this gene rebalancing. This hypothesis suggests a complex interplay between epigenetic modifications, transcriptional regulation, and lipid homeostasis in the context of ALS pathology.

PUFAs emerged as promising therapeutic candidates in ALS due to their neuroprotective and anti-inflammatory properties. Multiple large epidemiological studies showed that higher dietary intake and plasma levels of specific PUFAs, particularly alpha-linolenic acid, are associated with a lower risk of developing ALS, prolonged survival, and slowed functional decline in patients [151, 245, 246]. Experimental models, including Drosophila and human neuronal cultures, demonstrated that increasing neuronal PUFA levels, either through dietary supplementation or by enhancing endogenous synthesis, can reduce neuronal death and can significantly extend lifespan in ALS models. Mechanistically, PUFAs may exert their protective effects by modulating neuronal membrane composition, reducing neuroinflammation, and improving resilience to cellular stressors implicated in ALS pathogenesis [151]. However, the efficacy of dietary PUFA supplementation may be limited by challenges in delivering sufficient quantities to neurons, as the BBB restricts PUFA transport.

**Fig. 4:**
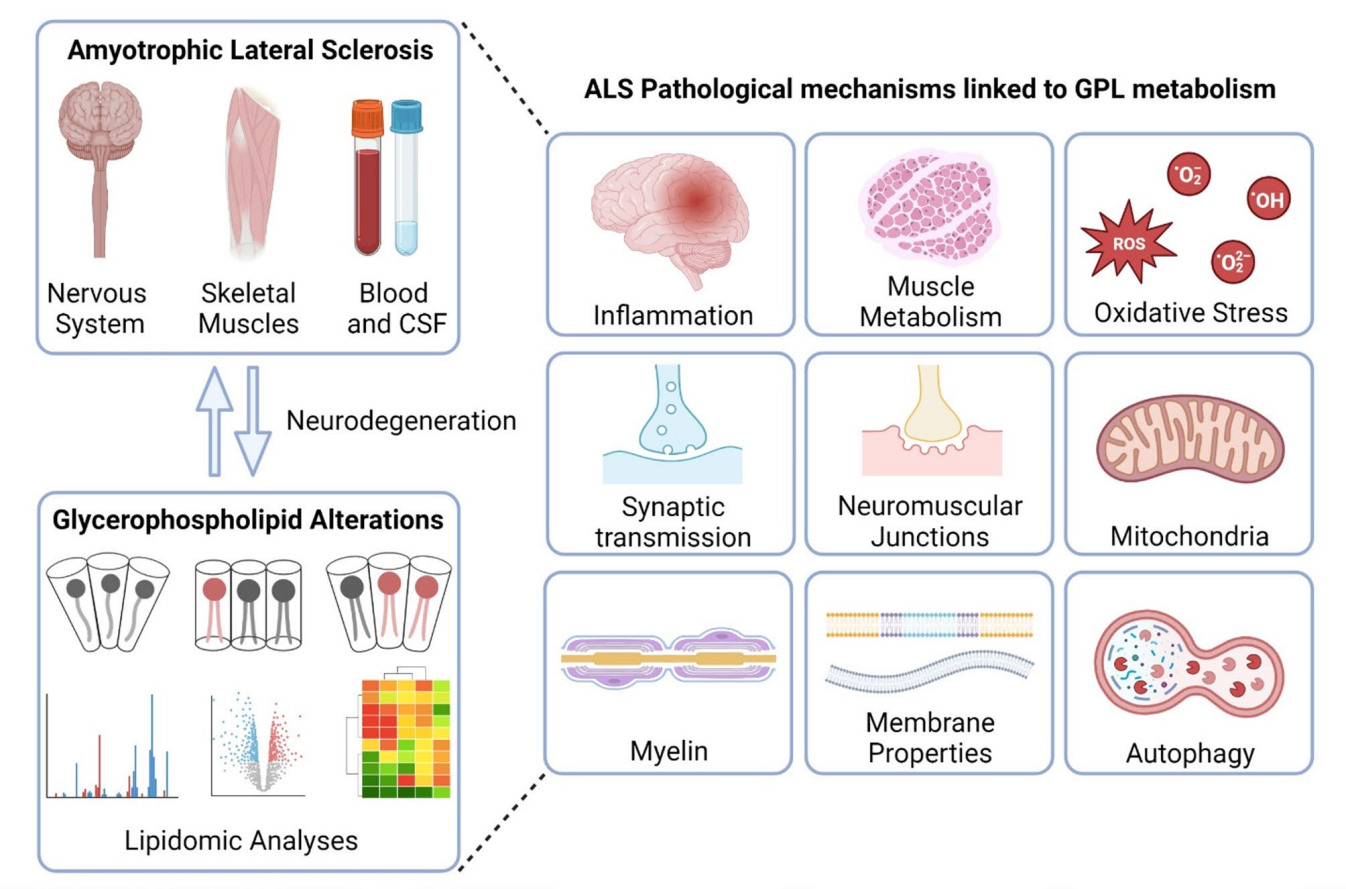
Link between GPL functions and ALS pathological mechanisms. Lipidomic analyses of the nervous system, skeletal muscles, and biofluids demonstrated the alterations of GPL metabolism. The perturbation of GPL homeostasis in ALS influences various pathological mechanisms, including inflammation, synaptic transmission, myelin disruption, muscle atrophy, neuromuscular junction dismantlement, membrane properties alterations, oxidative stress, mitochondrial defects, and autophagy dysfunctions. Created with Biorender.com

Currently, only a limited number of potential biomarkers and therapeutic targets related to GPL metabolism were identified, but they certainly hold promise for future research endeavors (Fig. 4). Modulation of GPL metabolism in ALS could have a beneficial effect on various affected physiological mechanisms such as autophagy, synaptic transmission, myelin formation, NMJ function and muscle metabolism. Further work exploring the link between GPL alterations and pathological mechanisms will hopefully reveal impactful insights.

### Current limitations and challenges

Lipidomic technologies have made significant advances in recent years, but several challenges persist. The limited availability of isotope-labeled internal standards for all lipid classes prevents accurate quantification and exhaustive mapping of entire lipidomes [234]. The identification and annotation of unknown lipids is still complex, with isobaric or isomeric masses often overlapping between species. This complexity is exacerbated by the high chemical diversity of lipids, necessitating a combination of different mass spectrometry methods for comprehensive analysis [234]. Besides, unidentified lipids are often disregarded in datasets, while they can be highly informative about pathological mechanisms. For instance, non-annotated lipids discovered in the plasma and CSF were among the most discriminant features between ALS patients and controls [20].

The lack of standardized protocols for sample preparation, data acquisition and analysis led to poor cross-platform reproducibility [234]. Unlike other omics technologies with relatively standardized protocols, lipidomics suffers from a disparity in methodologies and workflows, contributing to inter-study variability [247]. Additionally, integrating lipidomics data with other omics datasets remains challenging.

This variability is further exacerbated by the heterogeneous nature of ALS, where patients exhibit considerable discrepancies in age and site of onset, motor manifestations, degree of frontotemporal involvement, spreading patterns, and disease duration [1]. The interplay of genetics, environmental factors, and lifestyle choices is pivotal in shaping the disease trajectory and similar clinical phenotypes might arise from various factors, amplifying the intricacy of the disease pathology. These multiple layers of heterogeneity undoubtedly impact the lipidomic profile of ALS patients.

Many existing lipidomic studies suffer from limitations such as small sample sizes (*n* < 30), lack of statistical power, and missing relevant information on patient status. This is especially true for invasive procedures such as lumbar punctures and muscle biopsies. To address these issues, future studies should focus on improving statistical robustness using well-defined ALS patient cohorts with adequate controls. Furthermore, studies of homogeneous subpopulations regrouping similar gene mutations could increase the statistical power and elucidate the impact of specific mutated genes on lipid metabolism. Additionally, longitudinal follow-up of ALS gene carriers could unravel potential pre-symptomatic lipidomic alterations. It is crucial for studies to include comprehensive patient history, including functional scales like the ALSFRS score, and to match cohorts for factors influencing lipid levels, such as sex, age, and dietary habits.

In mice, these factors also influence directly the lipid profile of the animals, in addition to the genetic background. A comparative study of α-synuclein mouse models of Parkinson’s disease revealed age, gender, and gene dosage as factors modifying brain lipid profiles [248]. Additionally, the mouse lipidome could be influenced by laboratory-dependent practices such as the time of sample collection (influence of circadian rhythms) or the type of diet given to mice (influence of the percent of fat) [104, 249]. As a consequence, lipidomic data in ALS mouse models should be cautiously interpreted, especially because most of them come from mutant *SOD1* models with different genetic background and gene engineering methods.

A significant challenge in analyzing lipidomic data from brain tissue lies in the complexity of the samples, which encompass diverse regions and a variety of cell types. Given that glial cells outnumber neurons in most brain areas, the resulting lipid profiles are largely influenced by the glial metabolism. Despite this, the findings are often interpreted in the context of neuronal functions. Recent advances in mass spectrometry technologies enable cellular profiling at unprecedented resolution, down to the level of single cells and even individual organelles, facilitating the identification of cell-type-specific pathological mechanisms associated with lipid metabolism [250–252]. Neurons, astrocytes, oligodendrocytes, and microglia all have distinct lipidomes [253], and in vivo lipidomic alterations at the cellular level have not been investigated yet in ALS. So far, different cell types have been investigated separately in a dish. However, it is important to note that *in vitro* lipid profiles often contrast with in vivo lipidomes [254, 255]. In vitro mono-cultures lack the crucial interactions between neurons and glial cells, which are decisive in maintaining lipid homeostasis [256]. Besides, impaired communication leads to cortical neuron degeneration in hereditary spastic paraplegia (HSP) [257] and contributes to lipid dysregulation, bioenergetic deficits, and increased risk to develop Alzheimer’s disease in ApoE4 carriers [258]. Co-cultures, organoids/assembloids, and microfluidic devices offer the possibility of recreating the coupling of neuron-glial cells with a higher fidelity [198].

The cell culture medium also dramatically influences the lipid profile since it is generally not designed to maintain the in vivo cellular lipid composition. Cultured cells are often exposed to high glucose concentrations coupled with a lipid deficit, leading to accelerated rates of lipogenesis and higher rates of *de novo* fatty acid biosynthesis [254]. In standard culture mediums, the unique source of lipids is the added serum. Since vertebrate cells cannot produce PUFAs through *de novo* synthesis, cells in culture have a highly restricted range of PUFA levels in their membrane compared to cells in their in vivo environment [255]. The lack of PUFAs dramatically affects neural cells, which are highly enriched with these fatty acids in vivo [259]. PUFAs are mainly present in GPLs at plasma membranes. Their reduced availability could affect (1) membrane properties, such as fluidity, flexibility, and permeability (2), cell signaling and gene expression through decreased formation of bioactive molecules, and (3) cell oxidative status since PUFAs have a higher oxidizing capacity than MUFAs or SFAs. Although cellular fatty acid composition in vitro and in vivo differ, GPL composition is similar [255].

To advance the field, it is crucial to validate in vitro lipid-related findings in vivo models and to develop standardized protocols that account for the complex interplay of factors influencing lipidomic profiles in both research and clinical settings.

## Conclusions

Increasing pre-clinical and clinical data demonstrate consistently the involvement of GPL alterations in ALS disease pathology. Remarkably, some changes emerge during the early phase of the disease, suggesting their possible role in initiating neurodegeneration. Furthermore, these alterations persist and evolve at later stages and might exacerbate ALS pathology. Nevertheless, further studies are necessary to unravel the mechanistic link between GPL metabolism alterations and neurodegeneration. Future investigations will not only enhance our understanding of ALS pathogenesis but also offer novel diagnostic and prognostic biomarkers, paving the way for innovative therapeutic strategies. To achieve these goals, a comprehensive approach involving the joint analysis of multiple models, tissues, ans biofluids will be necessary to reveal the complete fingerprint of GPL alterations in ALS. In clinical settings, results must be validated across independent large-scale cohorts of patients with standardized procedures to ensure reliability and reproducibility. The recent findings underscore the critical importance of investigating GPLs in greater detail to fully harness their potential in ALS research and treatment.

## Data Availability

Not applicable.

## Abbreviations

AADHAPR: Acyl/Alkyl-dihydroxyacetone phosphate reductase
ADAPS: Alkyl-dihydroxyacetone phosphate synthase
AGK: Acylglycerol kinase
AGPAT: Acyl/Alkyl- glycerol-3-phosphate acyltransferase
ALS: Amyotrophic lateral sclerosis
ARA: Arachidonic acid
ASOs: Antisense oligonucleotides
BBB: Blood-brain barrier
BMI: Body mass index
CDS: Cytidine diphosphate-diacyglycerol synthase
CDP-DAG: Cytidine diphosphate-diacyglycerol
CK: Choline kinase
CL: Cardiolipin
CLS: Cardiolipin synthase
CPT: Choline phosphotransferase
CSF: Cerebrospinal fluid
CT: Phosphocholine cytidyltransferase
CTP: Cytidine triphosphate
DAG: Diacylglycerol
DGK: Diacylglycerol kinase
DHA: Docosahexaenoic acid
DHAP: Dihydroxyacetone phosphate
DHAPAT: Dihydroxyacetone phosphate acyltransferase
EK: Ethanolamine kinase
EPT: Ethanolamine phosphotransferase
ER: Endoplasmic reticulum
ET: Phosphoethanolamine cytidyltransferase
ETC: Electron transport chain
FTD: Frontotemporal dementia
G3P: Glycerol-3-phosphate
GPL: Glycerophospholipid
GPAT: Glycerol-3-Phosphate acyltransferase
GSK3β: Glycogen synthase kinase 3 beta
HDAC: Histone deacetylase
HSP: Hereditary spastic paraplegia
IP_3_: 1,4,5-trisphosphate
iPSCs: induced pluripotent stem cells
LMN: Lower motor neuron
LPA: Lysophosphatidic acid
LPAAT: Lysophosphatidic acid acyltransferase
LPC: Lysophosphatidylcholine
LPE: Lysophasphatidylethanolamine
LPI: Lysophosphatidylinositol
LPLAT: Lysophopsolipid acyltransferase
LPS: Lysophosphatidylserine
LTP: Lipid-transport protein
MAM: ER-mitochondria associated membrane
MN: Motor neuron
MUFA: Monounsaturated fatty acid
OPC: Oligodendrocyte progenitor cell
PA: Phosphatidic acid
PAP: Phosphatidic acid phosphatase
PC: Phosphatidylcholine
PE: Phosphatidylethanolamine
PEDS1: Plasmanylethanolamine desaturase
PG: Phosphatidylglycerol
PGP: Phosphatidylglycerol phosphate
PGPP: Phosphatidylglycerol phosphate phosphatase
PGPS: Phosphatidylglycerol phosphate synthase
PLA: Phospholipase A
PLB: Phospholipase B
PLC: Phospholipase C
PLCD1: Phospholipase C delta 1
PLD: Phospholipase D
PLS: Primary lateral sclerosis
PI: Phosphatidylinositol
PI(4,5)P_2_: Phosphatidylinositol 4,5-biphosphate
PIP_4_: Phosphatidylinositol 4-phosphate
PIS: Phosphatidylinositol synthase
PS: Phosphatidylserine
PSD: Phosphatidylserine decarboxylase
PSS: Phosphatidylserine synthase
PUFA: Polyunsaturated fatty acid
ROS: Reactive oxygen species
SERCA: Sarcoplasmic reticulum calcium ATPase
SFA: Saturated fatty acid
UMN: Upper motor neuron
